# Prevalence of body dissatisfaction and its effects on health-related quality of life among primary school students in Guangzhou, China

**DOI:** 10.1186/s12889-019-6519-5

**Published:** 2019-02-20

**Authors:** Wei Liu, Rong Lin, Chongshan Guo, Lihua Xiong, Siyu Chen, Weijia Liu

**Affiliations:** Faculty of School Health, Guangzhou Centre for Disease Control and Prevention, No. 1, Qide road, Jiahe, Baiyun District, Guangzhou, 510440 China

**Keywords:** Weight status, Health-related quality of life, Body dissatisfaction, Body image, Children

## Abstract

**Background:**

The relationship between body dissatisfaction (BD) and health-related quality of life (HRQoL) has been well documented in adolescents and adults but is less clear in children, particularly in China. The aims of this study were to describe body image perception and dissatisfaction and to examine their effects on HRQoL among primary school students in Guangzhou, China.

**Methods:**

A total of 5734 children aged 8–12 years from 29 schools completed self-report questionnaires, which included the Paediatric Quality of Life Inventory 4.0 for measuring HRQoL and Ma figural stimuli for measuring BD. Based on their level of BD, the children were divided into three groups: no dissatisfaction, mild dissatisfaction and moderate or high dissatisfaction. Based on the children’s perceptions of their own body image, the groups were also categorized into just right, too fat and too thin groups. Height and weight were objectively measured using standardized methods, and a BMI z-score was derived using the age- and sex-specific WHO references from 2007 for children aged 5–19 years. Weight status was classified as underweight, healthy weight, overweight or obese.

**Results:**

A total of 78.10% of children aged 8–12 years in Guangzhou had different levels of BD; boys had slightly higher levels BD than girls (*p* < 0.01), and obese children demonstrated the highest degree of BD (*p* < 0.01). However, BD levels did not differ significantly according to age (*p* = 0.194). Gender differences in body image perceptions were only found in children with a healthy weight (*p* < 0.01), but age differences in body image perception were present in both children with a healthy weight (*p* < 0.05) and underweight children (*p* < 0.05). Of the children with a healthy weight who were dissatisfied with their body image, 65.54% of the boys wanted to be heavier, whereas 52.95% of the girls wanted to be thinner (*p* < 0.01), and older children were more inclined to perceive themselves as too fat (*p* < 0.01). After controlling for the influence of confounding factors, significant trends for lower HRQoL scores with increasing BD levels persisted in all domains (*p* < 0.01).

**Conclusions:**

BD is as common in children as in adolescents and adults and might be independently associated with HRQoL impairment. The present findings suggest that the current epidemic of BD is a threat to the health of primary school children in China, and prevention programmes for this population should be implemented in the future.

## Background

Health-related quality of life (HRQoL) is a comprehensive and multi-dimensional construct that includes the self-assessment of physical, emotional and social well-being [1]. Numerous studies based on clinical samples have revealed that obesity can significantly impair HRQoL in children and adolescents [2–5]; for example, the HRQoL of severely obese children is comparable to the HRQoL of children with cancer [6]. However, the evidence supporting an association in population-based samples has remained inconclusive. A large number of relevant population-based studies have shown a significant inverse association between obesity and HRQoL, particularly in the physical, social and school domains [7–9]. However, other studies have found no associations between weight status and HRQoL [10–12].

Given these inconsistent results, researchers have begun to conduct in-depth studies to explore the underlying effects of obesity on HRQoL among children and adolescents. Recent studies have suggested that the effects of obesity on HRQoL can indeed vary by gender, age, race and culture, particularly in the psychosocial dimension. Stronger associations between overweight/obesity and poor HRQoL have been found among girls and older children than among boys and younger children, respectively [7, 9, 13]. Being overweight or obese can significantly reduce the HRQoL scores of white children, but not black children, and cultural differences might also be an important confounding factor in the relationship between overweight and HRQoL [9, 10].

Some studies have shown that body dissatisfaction (BD) is associated with impairments in various aspects of HRQoL [14–17]. BD, which refers to a person’s subjective dissatisfaction with his or her body size or shape, has been recognized as central to the health and well-being of children and adolescents who are overweight, and BD has become a public health problem [14, 17]. Because BD is widespread throughout the general population, particularly among adult women in Western countries, it was labelled “normative discontent” in the 1980s [18, 19]. Evidence has shown that BD is associated with low self-esteem, depressed mood and eating disorder symptoms [20–23]. BD plays a significant mediating role in the impact of obesity on HRQoL and has also been frequently reported in recent years, particularly in women and in psychosocial domains [16, 24–26]. Other studies have demonstrated that BD can independently affect HRQoL [14, 27]. In addition, Mond et al. even reported that impaired emotional well-being of overweight adolescents is due to BD rather than body weight per se; when the effects of BD are statistically controlled for, obesity is no longer associated with lower self-esteem or a more depressed mood [17].

To our knowledge, most studies have examined the relationship between BD and HRQoL in Western or developed countries, and the subjects are most often adolescents or adults, especially females [15, 24, 28–30]. The effects of BD on HRQoL among children living in lower-income countries, such as China, and those younger than 12 years have rarely been studied.

The primary purpose of the current research was to extend our previous analysis of the associations between weight status and HRQoL by examining the associations between BD and HRQoL in the same study populations [9] and to determine the prevalence of BD in this population.

## Methods

### Sampling and participants

The data were obtained from a larger cross-sectional study that aimed to determine the prevalence and risk factors for being overweight and obese in children aged 5–12 years [9, 31]. To obtain a representative sample, a multi-stage, stratified cluster random sampling method was adopted. Further details on the sampling methods and investigation processes have been previously reported [9]. A total of 29 primary schools (20 public and 9 private) from 10 urban districts in Guangzhou were selected. Written informed consent forms were disseminated for all of the children in grades 1 to 5 in the 29 selected schools (11,445 students; age 5–12 years old) to bring to their parents, and 9917 (86.6%) students agreed to participate.

Anthropometric measurements were obtained from all the participating children at school by trained research staff using standardized procedures and equipment, and the children’s parents were asked to complete a questionnaire that included questions about their demographic and family economic status. Given the inherent difficulties in collecting survey data from young children [32], only students in grades 3 and above (8+ years, *n* = 5962) were required to complete a student self-report questionnaire, which included measures of HRQoL and BD. After 228 (3.8%) incomplete student questionnaires were excluded, 5734 children aged 8–12 years were included in this analysis (96.2%).

### Height and weight measurements

As reported previously [9], the children wore light clothing with no shoes for the height and weight measurements. Height was measured to the nearest 0.1 cm using a TGZ-type height tester (Teaching Instrument and Equipment Co. Ltd., Dalian), and the weight was measured to the nearest 0.1 kg using an electronic scale (JH-1993T, Weighing Apparatus Co. Ltd., Dalian). Body mass index (BMI) was calculated (weight (kg)/height (m)^2^), and the standard deviation score (BMI z-score) was derived using the age- and sex-specific WHO references from 2007 for children aged 5–19 years. The children were also categorized as underweight (<-2SD), healthy weight (between -2SD to 1SD), overweight (between 1SD to 2SD) or obese (>2SD) [33]. The measuring devices were systematically calibrated.

### Measurement of body dissatisfaction

As the most commonly used tool for measuring BD, figural drawing scales have the advantage of quick administration and the ability to collect group data. Usually, these scales consist of a series of frontal images ranging from thin to fat. Participants are asked to select the image that most represents their current body size and ideal body size, and the discrepancy between these two ratings represents a measure of body dissatisfaction [34, 35]. Studies have shown that children can accurately estimate their body size using this instrument and can produce a valid measure of body satisfaction [36, 37].

In the current research, the Ma figural stimuli, which were developed from Collins’ (1991) stimuli and adapted for a Chinese population [38], were adopted to measure the children’s BD. The Ma figural stimuli consist of four arrays of pictures for children or adolescents and boys or girls, and only the two arrays for adolescents were used in this study. Each of the arrays contains seven drawings ranging from very thin to very fat [38]. The participants were asked to select one picture that best fit each of the following statements: (1) “Which picture looks most like you?” (for current body size), and (2) “Which picture shows the way you would like to look?” (for ideal body size). A scale from 1 to 7 was provided, and a higher score indicated a larger body size. Subtracting the ideal body size from the current ideal body size yielded the BD score, which ranged from − 6 to 6. Based on the BD scores, the participants were classified into three groups: (1) satisfied with their current body shape (BD = 0); (2) perceived themselves as too thin (BD < 0); and (3) perceived themselves as too fat (BD > 0). Based on the absolute values of the differences in BD scores, the participants were again classified into three groups: (1) satisfied with their current body shape (|BD| = 0); (2) mild dissatisfaction with their current body shape (|BD| = 1); and (3) moderate or high dissatisfaction with their current body shape (|BD| > 1).

### Measurement of HRQoL

The Chinese version of the PedsQL 4.0 was used to assess HRQoL in this study. This scale was developed using the standard procedure for cross-cultural adaptation under the authority of Varni and showed good reliability and validity, with Cronbach’s alpha values ranging from 0.74 to 0.82 for all of the subscales [39]. Detailed information about PedsQL 4.0 has been reported elsewhere [40]. In brief, the PedsQL is an instrument with 23 items grouped into four subscales: physical (8 items), social (5 items), emotional (5 items), and school (5 items) functions. Each item asks how much a particular behaviour had been a problem in the past month. Mean scores were calculated based on a 5-point response scale for each item and transformed to a 0–100 scale, with a higher score representing better quality of life. The PedsQL subscales can be used to derive three summary scores: a total score (mean of all items), a physical health score (mean of physical function items), and a psychosocial health score (mean of emotional, social and school function items).

### Collection of parental data

As reported previously [9], the parents were asked to complete a questionnaire that included questions about maternal and paternal education levels, maternal and paternal heights and weights (to calculate BMI), and family income.

### Statistical analysis

All statistical analyses were performed using SAS software, version 9.3. Descriptive statistics were used to summarize the demographic, anthropometric and HRQoL data. Independent-samples t-tests (two groups) or general linear models (three or more groups) were used to explore differences in HRQoL between subgroups. Pearson’s and Spearman’s correlation analyses were used to assess the relationship between age and HRQoL and the degree of BD, respectively. Wilcoxon’s test or the Kruskal-Wallis test was used to assess the differences in the degree of BD between subgroups (e.g., gender, weight status), and the χ^2^ test and Fisher’s exact test were used to assess the differences in body image perception between subgroups.

To explore the association between BD and HRQoL considering the effect of school as a cluster (z = 3.46, *p* < 0.001), multilevel random-effects models (individual level and school level) were developed with HRQoL scores (total, physical summary, psychosocial summary, emotional function, social function and school function scores) as the dependent variables and BD (grouped into no dissatisfaction (reference group), mild dissatisfaction, moderate or high dissatisfaction), gender and age as the independent variables. As in our previous analysis of associations between weight status and HRQoL in the same study population [9], other covariates (BMI, school type, maternal education, paternal education and family income) were included in the multilevel models if statistically significant differences in HRQoL were found between the subgroups in the univariate analyses. School was added as a random effect. The level of significance in the analyses was set to 0.05.

## Results

### Participant characteristics

The descriptive characteristics of the participants are shown in Table 1. The average age was 9.74 (SD = 1.01) years, and 54.48% were boys. The mean BMI z-score was − 0.13 (SD = 1.33, range − 4.01 to 5.51). Approximately 80% of the participants were dissatisfied with their body shape, with 52.63% reporting mild dissatisfaction and 25.46% reporting moderate or high dissatisfaction. Although only 27% of the participants had an unhealthy weight, 43.02% perceived themselves as too fat, and 35.07% perceived themselves as too thin.

**Table 1:** Comparison of unadjusted mean health-related quality of life (HRQoL) total scores among children with different demographic and socioeconomic characteristics

	n (%)	HRQoL total score Unadjusted mean (s.d.)	*P*
Total	5734 (100.00)	78.91 (13.56)	
Gender			
Male	3124 (54.48)	78.45 (13.87)	**0.005**
Female	2610 (45.52)	79.46 (13.17)	
Age (year)			
8	601 (10.48)	75.97 (14.16)	**< 0.001**
9	1906 (33.24)	78.57 (13.97)	
10	1746 (30.45)	79.26 (13.32)	
11	1332 (23.23)	80.47 (12.77)	
12	149 (2.60)	77.01 (13.29)	
School type			
Private school	1726 (30.10)	75.43 (13.76)	**< 0.001**
Public school	4008 (69.90)	80.41 (13.20)	
Weight category			
Underweight	373 (6.51)	77.87 (13.12)	0.476
Healthy weight	4187 (73.02)	78.97 (13.67)	
Overweight	751 (13.10)	78.92 (13.18)	
Obesity	423 (7.38)	79.21 (13.51)	
Body perception			
Just right	1256 (21.90)	82.01 (13.79)	**< 0.001**
Too fat	2467 (43.02)	78.18 (13.44)	
Too thin	2011 (35.07)	77.87 (13.29)	
BD			
Not at all	1256 (21.90)	82.01 (13.79)	**< 0.001**
Mild	3018 (52.63)	78.87 (13.04)	
Moderate or high	1460 (25.46)	76.32 (13.88)	
Paternal education years (*n* = 5407)			
< =9	2352 (43.50)	76.92 (13.66)	**< 0.001**
10–12	1619 (29.94)	79.50 (13.12)	
> 12	1436 (26.56)	81.92 (12.74)	
Maternal education years (*n* = 5416)			
< =9	2772 (51.18)	76.86 (13.63)	**< 0.001**
10–12	1346 (24.85)	80.39 (12.69)	
> 12	1298 (23.97)	82.24 (12.76)	
Family income (*n* = 5038)			
Low	1926 (38.23)	77.3 (13.83)	**< 0.001**
Medium	2072 (41.13)	79.43 (13.15)	
High	1040 (20.64)	81.54 (12.72)	

The level of significance in the analyses was set to 0.05, all *p* < 0.05 were used in boldface

As shown in Table 1, the unadjusted overall mean total HRQoL score was 78.91 (SD = 13.56); girls had a slightly higher score than boys (*t* = 2.84, df = 5732, *p* = 0.005), and age was positively associated with HRQoL (β = 0.07, *p* < 0.001). Students in public schools had much higher scores than those in private schools (*t* = 12.70, df = 5732, *p* < 0.001). Children who were dissatisfied with their body shape (including perceiving themselves as too fat and too thin) had significantly lower unadjusted total HRQoL scores than their peers (78.04 and 82.01, respectively, *t* = 9.08, df = 5732, *p* < 0.001). The greater the magnitude of a child’s dissatisfaction with his or her body shape, the lower his or her unadjusted total HRQoL score (*F* = 60.79, df = 2/5731, *p* < 0.001). Additionally, we found a significant correlation of years of parental education and family income with the children’s total HRQoL score. However, no significant differences in HRQoL were found between children with different weight statuses (*F* = 0.83, df = 3/5730, *p* = 0.476).

### Body image perception and dissatisfaction

As shown in Table 2, 78.10% of the children aged 8–12 years in Guangzhou had different levels of BD, and boys (51.25 and 28.81%, respectively) had slightly higher levels of BD than girls (54.29 and 21.46%, respectively) (*z* = 6.43, *p* < 0.001). The degrees of BD were significantly different across weight categories (χ^*2*^ = 608.49, df = 3, *p* < 0.001), but the correlation between age and degree of BD was not significant (β = − 0.02, *p* = 0.194). Obese children had the highest level of BD, and more than 70% had moderate or high BD. Children with a healthy weight had the lowest incidence and levels of BD, although 56.46 and 17.43% of these students had mild and moderate or high BD, respectively.

**Table 2:** BD among children with different weight statuses

Weight category	N	Not at all n (%)	Mild n (%)	Moderate or high n (%)
Total	5734	1256 (21.90)	3018 (52.63)	1460 (25.46)
Underweight	373	45 (12.06)	209 (56.03)	119 (31.90)
Healthy weight	4187	1093 (26.10)	2364 (56.46)	730 (17.43)
Overweight	751	96 (12.78)	344 (45.81)	311 (41.41)
Obesity	423	22 (5.20)	101 (23.88)	300 (70.92)

Table 3 demonstrates that gender differences in body image perception existed only in children with healthy weight (χ^*2*^ *=* 196.67, df = 2, *p* < 0.001). Among these, more boys perceived themselves as too thin, whereas more girls perceived themselves as too heavy.

**Table 3:** Gender differences in body image perception of among children with different weight statuses

Weight category	n	Male (*n* = 3124)			Female (*n* = 2610)			*χ* ^*2*^	*P*
		Just right n (%)	Too fat n (%)	Too thin n (%)	Just right n (%)	Too fat n (%)	Too thin n (%)		
Underweight	373	20 (11.90)	11 (6.55)	137 (81.55)	25 (12.20)	14 (6.83)	166 (80.98)	0.02	0.990
Healthy weight	4187	519 (24.47)	552 (26.03)	1050 (49.50)	574 (27.78)	884 (42.79)	608 (29.43)	196.67	**< 0.001**
Overweight	751	67 (13.76)	391 (80.29)	29 (5.95)	29 (10.98)	226 (85.61)	9 (3.41)	3.81	0.149
Obesity	423	17 (4.89)	322 (92.53)	9 (2.59)	5 (6.67)	67 (89.33)	3 (4.00)	0.88	0.644

The level of significance in the analyses was set to 0.05, all *p* < 0.05 were used in boldface

Among the children with a healthy weight and those who were overweight, the average ages of those who perceived themselves as too fat were 9.75 (SD = 1.04) and 9.87 (SD = 1.04) years, respectively, which was slightly older than those who perceived themselves as too thin (9.64 ± 1.00 and 9.47 ± 1.00, respectively) (*t =* 3.02/2.44, df = 3092/653, both *p* < 0.05). In children who were underweight or obese, no age differences in body shape perception were found.

### Body dissatisfaction and HRQoL

As shown in Table 4, after controlling for the influence of BMI, school type, parental education and other confounding factors, the effects of gender, age and BD on children’s HRQoL were statistically significant on most subscales. Compared with girls, boys had lower HRQoL scores overall (β = − 0.74, *p* = 0.047) and for the psychosocial summary (β = − 1.90, *p* < 0.01), social function (β = − 2.31, *p* < 0.01) and school function (β = − 4.13, *p* < 0.01) but higher scores for the physical summary (β = 1.45, *p* < 0.01). With increasing age, HRQoL scores showed an upward trend for all of the subscales. Compared with children with no BD, those with mild and moderate or high BD had generally lower HRQoL scores across all domains, and a significant trend towards lower HRQoL scores with increasing BD levels was also found.

**Table 4:** Multi-level random-effects models for the factors influencing PedsQL scores^a^

	Total PedsQL score			Physical summary score			Psychosocial summary score			Emotional function score			Social function score			School function score		
	*β*	*SE*	*p*	*β*	*SE*	*p*	*β*	*SE*	*p*	*β*	*SE*	*p*	*β*	*SE*	*p*	*β*	*SE*	*p*
Gender (female^REF^ vs. male)	−0.74	0.37	**0.047**	1.40	0.45	**< 0.01**	−1.90	0.40	**< 0.01**	0.81	0.57	0.152	−2.31	0.46	**< 0.01**	−4.13	0.48	**< 0.01**
Age (years)	1.11	0.19	**< 0.01**	1.00	0.23	**< 0.01**	1.18	0.20	**< 0.01**	0.73	0.29	**0.011**	1.52	0.23	**< 0.01**	1.30	0.24	**< 0.01**
BD (Not at all^REF^ vs. Mild)	−2.96	0.47	**< 0.01**	−2.18	0.56	**< 0.01**	−3.38	0.50	**< 0.01**	−4.04	0.71	**< 0.01**	−2.69	0.58	**< 0.01**	−3.45	0.60	**< 0.01**
BD (Not at all^REF^ vs. Moderate or high)	−5.54	0.56	**< 0.01**	−4.86	0.67	**< 0.01**	−5.93	0.60	**< 0.01**	−7.06	0.85	**< 0.01**	−5.81	0.69	**< 0.01**	−5.00	0.71	**< 0.01**

^a^Multi-level random-effects models were used for the multivariate analyses; PedsQL scores were used as dependent variables; gender, age, BD, BMI, school type, maternal education years, paternal education years, and family income were used as fixed effects; and school was used as a random effect. The level of significance in the analyses was set to 0.05, all *p* < 0.05 were used in boldface

## Discussion

Overall, the study revealed that the prevalence of BD among children aged 8–12 years in Guangzhou was 78.10%, with boys having slightly higher levels than girls. These findings are consistent with those of previous studies of adolescents and adults. To some extent, this finding supplements knowledge about the level of BD among children in developing countries, such as China. BD was independently associated with significant impairment in all domains of HRQoL among children aged 8–12 years in Guangzhou, and as the level of BD increased, the reduction in the HRQoL score became more pronounced.

This research showed that the level of BD in boys was slightly higher than that in girls, but there were no age differences. The results were inconsistent with previous studies indicating that BD was more common in female subjects and increased with age in adolescents [19, 24], but they generally concurred with those obtained by a study in Portugal, which indicated that BD exhibited a slight increase with age in youths aged 12–18 years, but not in youths between the ages of 10 and 12 years [16]. There are two potential explanations for this outcome. First, most of the evidence was obtained from studies based on adolescents or adults, whereas this study focused on children aged 8–12 years, who had poorer independent cognitive abilities. Studies have shown that parents’ dissatisfaction with their children’s body shape and negative comments about their children’s bodies are related to children’s dissatisfaction with their own bodies [41, 42]. Cultural differences might be another factor. Most Chinese people expect children to be heavy, particularly boys [9, 43], because it is considered a sign of health and prosperity. Most likely for these reasons, this study found that approximately half of the boys with a healthy weight perceived themselves as too thin, which was notably higher than the proportion of girls with a healthy weight who perceived themselves as too thin.

Regardless of gender, children with obesity demonstrated higher BD levels than their healthy-weight peers, which agrees with the results of previous studies [16, 23, 44]. Most of the underweight children wanted to be heavier (perceived themselves as too thin), and most of the overweight/obese children wanted to be thinner (perceived themselves as too fat). These findings are consistent with the report of Li et al., which found that approximately 70% of obese children wished to be thinner and that 57.0% of underweight boys and 66.7% of underweight girls wished to be heavier [38].

Among the healthy-weight children, more boys wished to be heavier, whereas more girls wished to be thinner. The gender difference was similar to those found in previous studies, which indicated that girls wished to be slimmer, whereas boys preferred a heavier and more muscular/built appearance [38, 45, 46]. A potential explanation for the gender differences in BD is that the ideal body shape, i.e., slim for girls and muscular for boys, is driven by mass media [23, 47].

Our findings showed that HRQoL among children aged 8–12 years in Guangzhou differed by gender, age, school type, BD, parental education years and family income, but there was no difference among weight statuses. These findings agree with those of some population-based studies but contrast with the results of several other studies based on clinical samples [3–5, 10, 11]; we have reported these findings elsewhere [9]. After controlling for all of the confounding factors, including school type, BMI, parental education years and family income, higher BD levels were still associated with impaired HRQoL scores in all domains, which suggests that BD is not only an intermediary factor in the impact of weight status on HRQoL, as some researchers have reported [16, 26], but is also independently associated with HRQoL impairments. This finding was consistent with some previous studies [14, 27, 30] and presents some important information for researchers and policy makers who investigate public health initiatives addressing these issues in developing countries. In the future, it might be possible to improve children’s body image perceptions by strengthening relevant health promotion efforts and reducing the BD-related impairment of HRQoL [17, 24].

The large representative sample of children used to explore the relationship between childhood BD and HRQoL in China was a strength of this study. Additionally, the findings are generalizable to other Chinese urban populations (such as those of Beijing and Shanghai) because they were drawn from a large representative sample of both resident and migrant communities. A limitation of this study lies in the nature of cross-sectional studies themselves, which prevents the exploration of a causal relationship between BD and quality of life. Further studies are required to validate the current results and their potential explanations in China and other developing countries.

## Conclusions

This study demonstrated that the prevalence of BD among children aged 8–12 years in China is as high as it is in Western countries and that BD is significantly correlated with impaired HRQoL in this population. To reduce the occurrence of BD in primary school children, health promotion approaches may be taken in the future to improve their HRQoL.

## Abbreviations

BD: Body dissatisfaction
BMI: Body mass index
HRQoL: Health-related quality of life
SD: Standard deviation
SE: Standard error
WHO: World Health Organization
